# HLA-G as a prognostic marker in stage II/III colorectal cancer: not quite there yet

**DOI:** 10.1007/s00418-022-02141-w

**Published:** 2022-07-28

**Authors:** Alessandro Gambella, Stefano Scabini, Gabriele Zoppoli, Annalisa De Silvestri, Simona Pigozzi, Michele Paudice, Michela Campora, Roberto Fiocca, Federica Grillo, Luca Mastracci

**Affiliations:** 1 Department of Medical Sciences, University of Turin, Turin, Italy; 2 Oncological Surgery, IRCCS Ospedale Policlinico San Martino, Genoa, Italy; 3 Department of Internal Medicine, University of Genoa, Genoa, Italy; 4 IRCCS Ospedale Policlinico San Martino, Genoa, Italy; 5 Servizio di Epidemiologia Clinica e Biometria Direzione Scientifica-Fondazione Istituti di Ricovero e Cura a Carattere Scientifico (IRCCS) Policlinico San Matteo, Pavia, Italy; 6 Anatomic Pathology Unit, Department of Surgical Sciences and Integrated Diagnostics (DISC), University of Genoa, Genoa, Italy; 7 Anatomic Pathology, Ospedale Santa Chiara, Largo Medaglie d’Oro, 9, 38122 Trento, Italy

**Keywords:** Colorectal cancer, HLA-G, Immune response, Survival outcomes

## Abstract

Identifying innovative molecules involved in the tumor immune escape process could help refine the survival stratification of colorectal cancer (CRC) patients. HLA-G, a non-classical HLA molecule, physiologically involved in tolerogenic mechanisms, has recently emerged as a relevant prognostic marker in other tumor types, but ambiguous data are reported in the CRC setting. This study aims to evaluate the HLA-G expression and prognostic potential in a series of stage II/III CRCs. HLA-G expression was evaluated in 100 pT3 CRC cases by means of immunohistochemistry using the 4H84 and MEM-G/2 monoclonal antibodies. We observed heterogeneous expression of HLA-G showing different ranges: 4H84 expression ranged from > 1 to 40%—median 7%; MEM-G/2 expression ranged from 20 to 90%—median 50%. HLA-G positivity (any intensity > 1%) varied according to the antibody employed, identifying: 8 4H84 positive, 34 MEM-G/2 positive, 6 double-positive and 52 negative cases. Correlation with clinico-pathologic data showed a significant association with a poor tumor differentiation in stage III right-sided CRC subgroup (*p* = 0.043), while no other pathologic variable was significantly associated. Survival analysis revealed a reduced disease-free survival rate (HR 4.304613; *p* = 0.031) in the subgroup of CRC-related death cases, while no correlations were observed considering the whole series and the overall survival. In conclusion, HLA-G is a promising CRC prognostic marker however much work is still required regarding technical aspects and evaluation of expression.

## Introduction

Colorectal cancer (CRC) is the most frequent gastrointestinal malignancy, characterized by increasing incidence and heterogeneous survival outcomes (Ahadi et al. 2021; Gonzalez et al. 2021; Nagtegaal et al. 2020) with 5-year survival rate ranging from almost 90% in localized disease to 14–20% in advanced/systemic conditions (Petrelli et al. 2017; Renouf et al. 2013). A considerable survival gap exists between TNM stages II and III and even between patients grouped within the same stage (Edge and Compton 2010; Renouf et al. 2013; Shia et al. 2012). In this setting, innovative prognostic and predictive markers, which better identify survival, are emerging. These include serum biomarkers, molecular targets and tissue-tethered pathology features (Ferrando et al. 2020; Pitto et al. 2020; Remo et al. 2012).

In particular, the modulation of the immune response constituting the peritumoral environment represents a relevant feature of several neoplastic conditions, including CRC (Karpinski et al. 2017; Roelands et al. 2017). HLA-G is a non-classical variant of the HLA class I molecule family. It is a heterodimer composed of a heavy chain and a light chain [β2-microglobulin (β2M)], presenting four dimeric membrane-bound (HLA-G1, -G2, -G3 and -G4) and three monomeric soluble subtypes (HLA-G5, -G6 and -G7), encoded starting from alternative splicing of *HLA-G* gene located on chromosome 6p22.1 (Alegre et al. 2014). HLA-G is physiologically expressed in fetal and adult tissues, where it induces an immune tolerant environment by dampening the immune system response through the interaction with immune cell inhibitory receptors; specifically, HLA-G upregulation inhibits B and T cell proliferation, NK cytotoxicity function and neutrophil phagocytosis (Alegre et al. 2014; Carosella et al. 2000, 2015). Due to this peculiar immune inhibitory function, HLA-G overexpression also represents a pivotal immune escape mechanism of several malignancies (Lin and Yan 2015; Murdaca et al. 2018; Peng et al. 2021; Rouas-Freiss et al. 2014). However, contrasting data have been reported regarding the role of tumor cell HLA-G expression in the CRC setting (Fukushima et al. 1998; Guo et al. 2015; Kaprio et al. 2021; Reimers et al. 2014; Swets et al. 2016; Ye et al. 2007; Zeestraten et al. 2013; Zhang et al. 2017).

This study aims to evaluate the expression of HLA-G in a selected series of stage II and III CRC to identify its potential correlation with clinical, pathologic and survival data and, eventually, assess its role as a prognostic marker.

## Materials and methods

### Data retrieval

This is a non-consecutive retrospective study based on the clinical and pathology data of 100 CRC cases treated at the Policlinico San Martino Hospital, Genoa, Italy. All cases were pT3 CRC surgically resected at our institution’s Oncologic General Surgery Unit and selected to equally represent CRC site (right vs. left side) and stage (stage II vs. stage III). Patient’s demographic (e.g., gender, age at diagnosis), clinical [e.g., overall survival (OS), disease-free survival (DFS), cause of death] and pathology (e.g., tumor site, number of harvested lymph nodes, lymph node involvement) data were retrieved from the original medical records, regional civil registry office and pathology reports.

The study was conducted according to the guidelines of the Declaration of Helsinki. Patient consent was waived because of the retrospective nature of the research protocol and considering that it had no impact on patients’ care.

### Pathology analysis

Original slides were retrieved from the archives of the Pathology Unit and assessed to identify and select representative formalin-fixed paraffin-embedded tissue blocks. Representative blocks were then sectioned to obtain three 4-micron-thick sequential sections used for hematoxylin-eosin (H&E) and immunohistochemistry (IHC) stains. An experienced gastrointestinal pathologist analyzed H&E slides to review histopathologic features, such as CRC grading, infiltrative vs. expansile pattern, perineural and vascular invasion, poorly differentiated clusters and mucinous features.

HLA-G IHC stains were performed using the BenchMark XT AutoStainer^®^ (Ventana Medical Systems, Arizona, USA) according to the manufacturer’s instructions, as previously reported (Bragoni et al. 2017; Gambella et al. 2017). HLA-G IHC was performed using two clones of HLA-G, the 4H84 mouse monoclonal primary antibody (EXBIO Antibodies, Prague, Czech Republic) and the MEM-G/2 mouse monoclonal primary antibody (Abnova, Taipei, Taiwan).

Both antibodies were commercially available and were selected based on previous studies available in the literature. Normal placenta was used as positive control and normal colonic mucosa as negative control. Furthermore, for each immunohistochemical run, one slide was stained in the absence of the primary antibody to exclude the presence of background staining.

### HLA-G evaluation

All immunohistochemistry was assessed by expert pathologists (AG, FG, LM) independently, and any discordances were resolved by consensus. The percentage of cytoplasmic expression of HLA-G, of any intensity, was noted in tumor cells, and cases were evaluated as positive when expression was > 1%. HLA-G expression pattern was membranous and cytoplasmic. Patterns and sites of expression were noted (homogeneous vs. heterogeneous; invasive edge vs. tumor center).

### Statistical analysis

The Biometric and Medical Statistics Service of the San Matteo Hospital of Pavia, Italy, performed statistical analysis. Comparisons between groups were explored using Pearson’s chi-squared (*χ*^2^) test, Fisher test, Bartlett test and Kruskall-Wallis test, as appropriate. DFS and OS were calculated from the date of diagnosis to disease recurrence and death, respectively, and cases were censored at the last follow-up date if lost. Survival analyses were performed with the Kaplan-Meier method, and confounder impact was assessed with univariable Cox regression. The regression model for competing risks was used to explore variable correlation according to specific death causes, and the relative hazard ratio (HR) was estimated. Results were considered statistically significant for *p* value < 0.05.

### Image acquisition

Images were acquired by Leica DM 2000 microscope (Leica Microsystems, Wetzlar, Germany), with 60 × magnification objective lens in combination with a Leica Flexacam C1 (Leica Microsystems, Wetzlar, Germany) 12-MP stand-alone microscope camera and captured by Software On-screen display (OSD) for stand-alone operation LAS X for Windows. Image manipulation (adjustments of brightness, contrast and color balance) were applied when needed to the entire image.

## Results and discussion

In this study, we analyzed the role of HLA-G immunoexpression in a monocentric series of stage II/III pT3 CRCs using two commercially available antibodies. Our series consisted of 100 stage II and III pT3 CRC cases equally representing tumor site (50 right-side and 50 left-side CRC) and stage (50 stage II and 50 stage III CRC cases). Additionally, cases were further equally stratified combining these two features as 25 stage II and 25 stage III right-sided cases and 25 stage II and 25 stage III left-sided cases were collected. The mean age at diagnosis was 72.9 years, male-to-female ratio was 1:1, and most of the collected lesions presented low-grade differentiation according to WHO 2019 (90 cases). Overlapping intestinal bowel disease (IBD), vascular invasion, perineural invasion, poorly differentiated clusters and mucinous features were observed in the minority of cases (7, 14, 6, 20 and 34 cases, respectively). Of note, the seven cases with IBD were represented by ulcerative colitis (5 cases) and pancolitis (2 cases). Clinico-pathologic data of our series are detailed in Table 1.

**Table 1:** HLA-G expression and correlation with clinico-pathologic features

Climico-pathologic features	HLA-G expression					*p* value
	No expression	4H84 only	MEM-G/2 only	Both clones	Total	
Site						
Right colon	29	3	16	2	50	0.577
Left colon	23	5	18	4	50	
Growth pattern						
Expansile	24	4	23	2	53	0.182
Infiltrative	28	4	11	4	47	
Differentiation						
Low grade	49	6	30	5	90	0.475
High grade	3	2	4	1	10	
Inflammatory bowel disease						
Yes	47	8	32	6	93	0.645
No	5	0	2	0	7	
Vascular invasion						
Yes	45	6	30	4	86	0.573
No	7	1	4	2	14	
Pernineural invasion						
Yes	50	7	31	5	94	0.468
No	2	0	3	1	6	
Poorly differentiated clusters						
Yes	12	1	5	2	20	0.604
No	40	7	29	4	80	
Mucinous component^a^						
Yes	5	1	5	1	12	0.877
No	47	7	29	5	88	
Stage						
II-A (pT3N0)	27	5	15	3	50	0.715
III (pT3 N+)	25	3	19	3	50	

^**a**^Mucinous component ≥ 50% of tumor surface

**Fig. 1:**
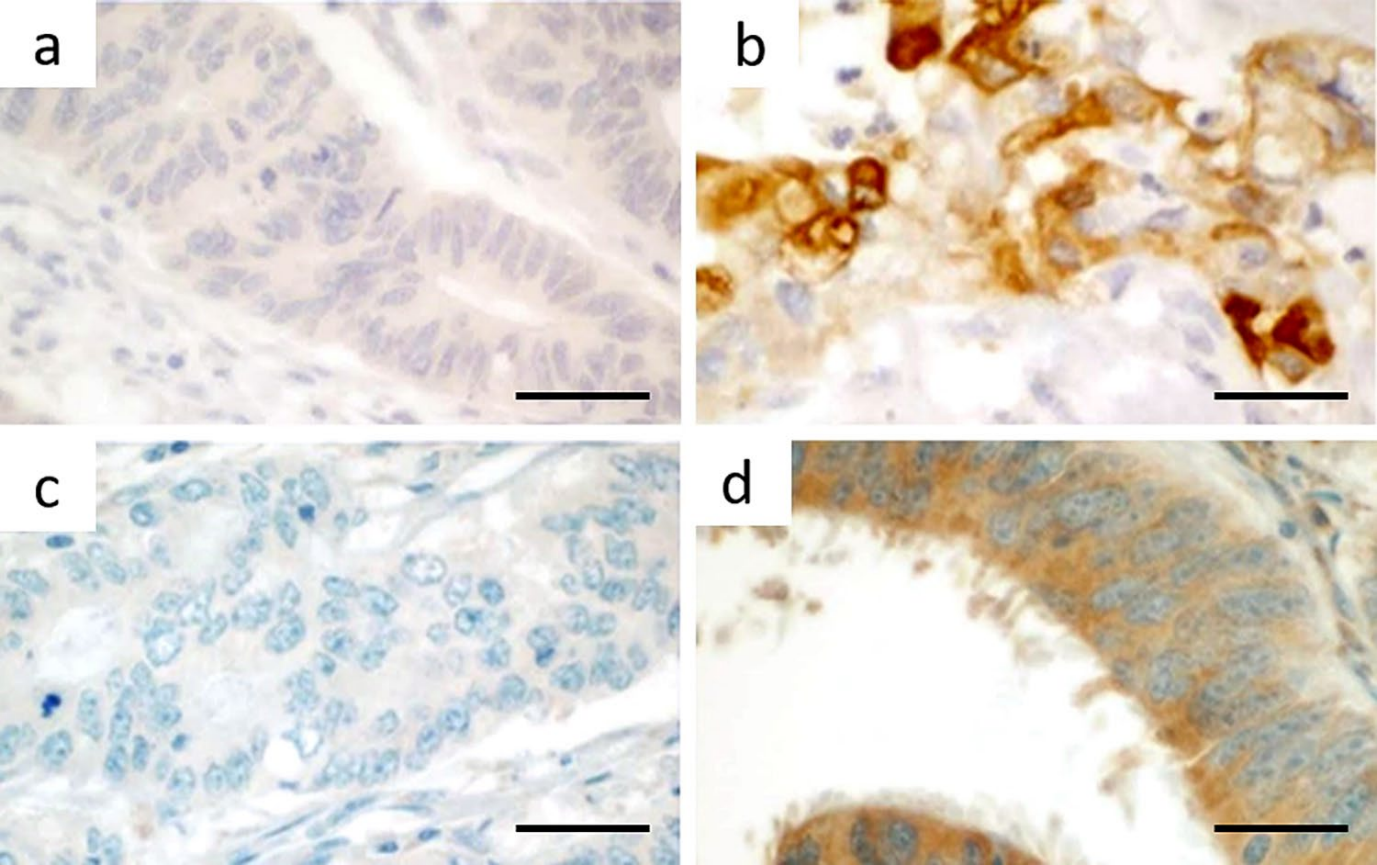
HLA-G immunoexpression according to the tested clones. **A** 4H84-negative case (original magnification 600×); **B** 4H84-positive case showing a cytoplasmic expression with membrane reinforcement (original magnification 600×); **C** MEM-G/2-negative case (original magnification 600×); **D** MEM-G/2-positive case showing cytoplasmic expression but lacking membrane reinforcement (original magnification 600×). All scale bars: 50 microns

HLA-G expression (Fig. 1) was heterogeneous in all cases, with greater heterogeneity seen with 4H84 clone. Comprehensively, the 4H84 clone showed a low percentage of positivity, ranging from 1% of cells to a maximum of 40% of cells in positive cases, with median percentage of expression of 7% in 14 cases. Regarding MEM-G/2 antibody, percentage of HLA-G expression was much higher, ranging between 20 and 90%, with median percentage of expression of 50% in 41 cases. Furthermore, no differences in site of expression between the invasive edge and the tumor center were seen.

In particular, HLA-G expression varied depending on the clone utilized: 8 cases were 4H84 positive and MEM-G/2 negative, 34 were 4H84 negative and MEM-G/2 positive, 6 were positive to both clones and 52 were negative to both clones. Subsequently, assessment of whether HLA-G expression (grouped into negative vs. positive with no clone-specificity distinction) correlated with any of the analyzed variables was undertaken, considering positivity for one antibody or the other, both or any positivity whatsoever. None of the associations with clinic-pathologic parameters was statistically significant (Table 1).

To further depict HLA-G expression across CRC site and stage, we stratified our series into four groups as follows: Group A (stage II right-sided CRC), Group B (stage III right-sided CRC), Group C (stage II left-sided CRC) and Group D (stage III right-sided CRC). Each group was composed of 25 cases and was queried to assess HLA-G correlation with pathology features. The only feature associated with increased HLA-G expression was poor differentiation within Group B (*p* = 0.043) (Table 2) when considering any positivity for any marker.

**Table 2:** HLA-G expression and its correlation with pathology features

			HLA-G expression				*p* value
			No expression	4H84 only	MEM-G/2 only	Both clones	
Group A	Grading	Low grade	16	1	5	1	0.828
		High grade	1	0	1	0	
	Growth pattern	Expansile	11	0	6	0	0.067
		Infiltrative	6	1	0	1	
	IBD	Absent	14	1	6	1	0.658
		Present	3	0	0	0	
	Vascular invasion	Absent	16	0	6	1	0.807
		Present	1	0	0	0	
Group B	Grading	Low grade	11	0	8	1	**0.043**
		High grade	1	2	2	0	
	Growth pattern	Expansile	4	1	5	0	0.708
		Infiltrative	8	1	5	1	
	IBD	Absent	10	2	8	1	0.875
		Present	2	0	2	0	
	Vascular invasion	Absent	10	2	8	0	0.205
		Present	2	0	2	1	
	Perineural invasion	Absent	11	2	9	1	0.957
		Present	1	0	1	0	
Group C	Growth pattern	Expansile	4	2	7	1	0.414
		Infiltrative	6	2	2	1	
	Vascular invasion	Absent	9	3	9	2	0.461
		Present	1	1	0	0	
Group D	Grading	Low grade	12	1	8	1	0.376
		High grade	1	0	1	1	
	Growth pattern	Expansile	5	1	5	1	0.622
		Infiltrative	8	0	4	1	
	Vascular invasion	Absent	10	1	7	1	0.782
		Present	3	0	2	1	
	Perineural invasion	Absent	12	1	7	1	0.417
		Present	1	0	2	1	

Cases were stratified in four groups as follows: Group A (stage II right-side CRC), Group B (stage III right-side CRC), Group C (stage II left-side CRC) and Group D (stage III right-side CRC)

Features absent in any group are not reported in the table, e.g., IBD cases in groups C or D

*p* value < 0.05 was considered significant (in bold)

**Fig. 2:**
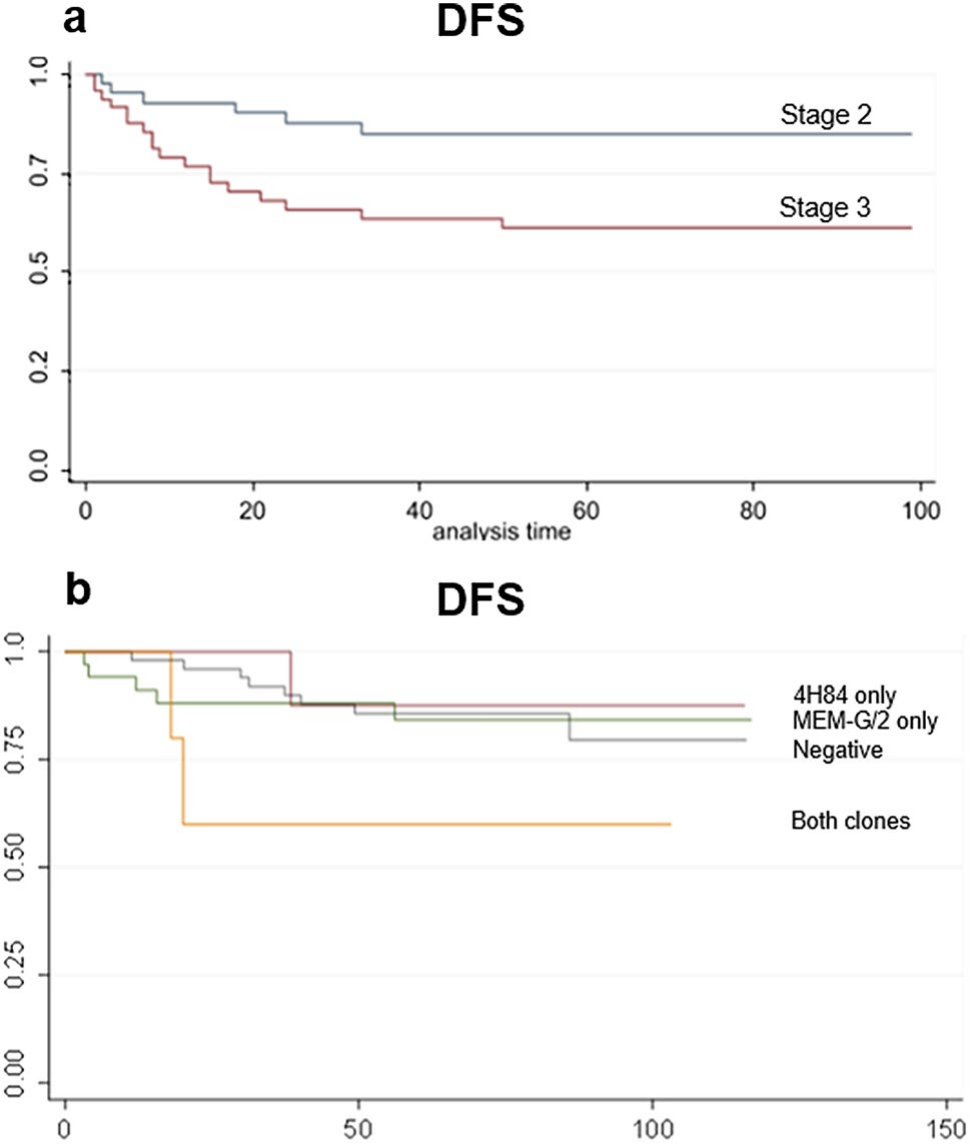
Survival analysis. **a** Disease-free survival differences according to stage (stage II vs. stage III). **b** Disease-free survival differences comparing HLA-G positive (stratified according to antibody clone) and negative cases

The mean follow-up period was 5.6 years. Overall, 36 patients died from any cause, of which 16 were CRC-related deaths. We evaluated the impact of stage on survival and observed that DFS was increased in stage II compared to stage III cases (*p* = 0.019) (Fig. 2a), whereas OS difference among stages (considering both CRC-related and non-specific death) was not statistically significant (*p* = 0.14).

In relation to survival, HLA-G expression was shown to significantly correlate with reduced DFS (HR 4.305; *p* = 0.031) in the subgroup of CRC-related death cases (Fig. 2b) when immunoexpression was identified with both clones; positivity with only one clone or the other did not impact on DFS (4H84 clone: HR 1.453, *p* = 0.568; MEM-G/2 clone: HR 0.8355, *p* = 0.709). No additional significant impact was observed regarding OS, considering either single vs. both clones in the overall series and the specific CRC-related death subgroup.

Our findings suggest that HLA-G negatively impacts disease-free survival, leading us to hypothesize a possible role of HLA-G as a prognostic marker of CRC.

The study has however underlined problems in the evaluation of HLA-G by immunohistochemistry regarding which antibody is used, specificity and cutoff ranges as well as the heterogeneity of population and samples used, making comparison between studies difficult.

The upregulated expression of HLA-G and its correlation with a worse clinical outcome have been established in several malignancies (Murdaca et al. 2018; Peng et al. 2021; van de Water et al. 2021). However, whether HLA-G harbors a prognostic value in the CRC setting is still debated, as summarized in Table 3. Favoring HLA-G’s harmful impact on CRC patient prognosis, three studies have shown a significantly reduced OS (HR 3.14, *p* = 0.021; HR 0.311, *p* = 0.008 and HR 1.428, *p* = 0.044 respectively) in the HLA-G positive subgroup (Ye et al. 2007; Guo et al. 2015; Zhang et al. 2017). On the other hand, three different studies did not identify any impact of HLA-G expression on patient prognosis (Fukushima et al. 1998; Kaprio et al. 2021; Zeestraten et al. 2014). A peculiar finding was that of Reimers et al. who demonstrated that patients with rectal cancer strongly expressing HLA-G showed prolonged DFS (HR 0.75; *p* = 0.042) compared to the 350 cases with a weak HLA-G expression (Reimers et al. 2014). In our study, HLA-G expression only partially affected patient survival. Indeed, among all the analyses performed, HLA-G expression significantly reduced the DFS in CRC-related death cases. However, widening the analysis to the overall series and both DFS and OS, HLA-G showed no prognostic value.

**Table 3:** Comparison between prognostic impact of HLA-G on colorectal cancer in different studies with focus on methodology

	Study population (*n*)	Stage	Rectal site (%)	Tissue evaluation	Antibody clone	% Cutoff	HLA-G positivity	Prognostic impact	OS/DFS/CSS
Fukushima et al. (1998)	39	I–IV	5% (no NAT performed)	Whole slide	Non-commercial polyclonal	n.a.	87.2%	Not evaluated	
Ye et al. (2007)	201	I–IV	60% (no NAT performed)	Whole slide	Non-commercial polyclonal	> 25%	65%	Negative impact	OS
Reimers et al. (2014)	484	I–IV	100% (no NAT performed)	TMA	4H84	Percentage and intensity (moderate intensity > 70% or strong intensity > 20%)	27.7%	Positive impact	DFS
Zeestraten et al. (2014)	285	n.a.	0% (rectal excluded)	TMA	4H84	Any intensity, any percentage	20.3%	No impact	OS/DFS
Guo et al. (2015)	102	I–IV	25.5% (NAT n.a.)	Whole slide	MEM-G/2	n.a.	70.6%	Negative impact	OS
Swets et al. (2016)	81	IV	n.a.	Whole slide	HLA 4H84	n.a.	29%	Not evaluated	
					MEM-G/1	n.a.	6%		
					MEM-G/2	n.a.	10%		
Zhang et al. (2017)	457	I–IV	49% (NAT n.a.)	Whole slide	4H84	> 5%	70.7%	No impact	OS
						> 55%	59.7%	Negative impact	
Kaprio et al. (2021)	307	I–IV	54% (NAT n.a.)	TMA	MAI-19219	n.a.	20.40%	No impact	CSS
Present study	100	II–III	7% (no NAT performed)	Whole slide	HLA 4H84	> 1%	14%	Negative impact	DFS
					MEM-G/2	> 20%	41%		
					HLA 4H84 + MEM-G/2		6%		

*n* number, *NAT* neoadjuvant treatment, *n.a.* not available, *TMA* tissue microarray, *OS* overall survival, *DFS* disease-free survival, *CSS* cancer-specific survival

What are the reasons behind the variable results for prognostic impact of HLA-G expression in the CRC panorama? The first problem in comparing different studies is the nature of the study populations collected. Numbers of CRCs studied vary considerably; indeed, most studies are relatively small with all stages considered (and this could affect HLA-G expression if it is recruited as an additional tumor escape mechanism in the later stages of the cancerogenetic sequence). Furthermore, sidedness has not, up to now, been considered, and our study is the first to actively distinguish between right and left sided CRCs; for example, previous contributions have had varying percentages of rectal cancers (5–100%) making up the case series, some with little information regarding any neoadjuvant treatment. A more in-depth analysis on cancerogenetic mechanisms (such as microsatellite instability [Remo et al. 2016; Kloor et al. 2010], which induces tumor neoantigen production and immune escape) and factors influencing immune response regulatory mechanisms and the peri-tumoral immune environment may lead to interesting results.

Another possible explanation for differences in survival could be based on the antibody used to assess HLA-G expression. In our study, HLA-G IHC expression did not perfectly match for cases comparing the two tested clones, namely 4H84 (which specifically recognizes the α1 domain of β2M-free HLA-G, but does cross-react with classical HLA-I) and MEM-G/2 (which binds a non-defined site of HLA-G heavy chain). Of note, Lin et al. reported a similar discordant IHC expression addressing HLA-G positivity when using 4H84 and 5A6G7 clones (Lin et al. 2018). They explained their heterogeneous findings based on the data of Tronik-Le Roux et al. that identified innovative HLA-G subtypes without α1 domain in CRC samples (Tronik-Le Roux et al. 2017). Further confirming this hypothesis, Swets et al. demonstrated the somehow contrasting data of HLA-G expression comparing Western blot and IHC analysis and within IHC analysis using different clones (Swets et al. 2018). Indeed, the recently identified “atypical” HLA-G isoforms could present different domain configurations leading to the heterogeneous expression observed so far and diluting the prognostic potential of HLA-G expression (Apps et al. 2008). As heterogeneity is a major problem in HLA-G assessment (at least for some commercially available antibodies), studies using tissue microarrays (TMA), and not whole slide evaluation, could cause significant biases in results.

Lastly, modalities of identification of expression (intensity vs. percentage of positivity vs. both) can also impact results. As exemplified in Table 3, cutoff values are not standardized (and often not available), and this may lead to greatly different percentages of HLA-G positivity in CRC (ranging from 6 to 87%). Many of the limitations introduced above are, unfortunately, also present in our contribution as the small sample size and the retrospective nature of our study must be stated.

In conclusion, considering (1) the well-defined role of the immune response in influencing CRC patient prognosis in both early localized lesions and advanced/metastatic disease, (2) the heterogeneous HLA-G IHC expression observed among different studies and adopting different clones, (3) the potential (unexplored) isoforms of HLA-G and (4) the different functions of HLA-G depending on its structural domains (Arns et al. 2020; Clements et al. 2007), the prognostic role of HLA-G in the CRC setting remains ambiguous and stokes the need for further, larger and potentially prospective studies.

## Data Availability

The data presented in this study are available on request from the corresponding author.
